# Facial pore refining by targeting dermal and epidermal functions: Assessment across age and gender

**DOI:** 10.1111/jocd.16424

**Published:** 2024-07-31

**Authors:** Morgane De Tollenaere, Marie Meunier, Anaïs Durduret, Emilie Chapuis, Pascale Auriol, Daniel Auriol, Amandine Scandolera, Romain Reynaud

**Affiliations:** ^1^ Givaudan Active Beauty, Research and Development Givaudan France SAS Pomacle France; ^2^ Givaudan Active Beauty, Research and Development Givaudan France SAS Toulouse France

**Keywords:** collagen, epidermis, extracellular matrix, pores, skin

## Abstract

**Background:**

Conspicuous facial pores are benign but represent a cosmetic concern for men and women. Recent works described dermal and epidermal impairments as clinical causes of enlarged pores. Morphological modifications of skin at the site of pores were associated with collagen density loss, possible alteration of extracellular matrix and abnormal differentiation of keratinocytes.

**Aims:**

A composition containing mannose‐6‐phosphate (Active Complex) was designed to address these different aspects of pore enlargement. In vitro and ex vivo evaluations were conducted in different models mimicking disturbance of dermal and epidermal functions. The pore refining activity of Active Complex was assessed in two clinical trials studying a Caucasian women cohort and an Asian men cohort.

**Results:**

At the dermal level, Active Complex upregulated collagen I and decorin synthesis, and genes encoding collagens I, III, V, VII, XVII; suggesting its ability to favor collagen fiber organization and anchorage. The downregulation of matrix metalloprotease, involved in extracellular matrix degradation, reinforced the protective effect of Active Complex in the dermis. Active Complex down modulated differentiation markers in keratinocytes as well as genes involved in cell renewal. Study of reconstructed human epidermis modeling keratinocyte hyperproliferation revealed that Active Complex mitigated two markers of this state: number of nuclei in the stratum corneum and involucrin expression. Clinical trials confirmed the pore refining activity of Active Complex on men and women of different ages and ethnicities; −24% total skin pore area after 56 days of application on women, and −30.2% on men after 7 days.

**Conclusions:**

This work demonstrates the interest to target dermal and epidermal modifications described in conspicuous pore area, especially dermis fiber organization, to address this cosmetic concern.

## 1 INTRODUCTION

Facial pores are microtopographic depressions at the skin surface, also described as funnel‐shaped structures, and corresponding to enlarged pilosebaceous follicles.^1^ Individuals commonly show visible pores, particularly in the nose, forehead, and cheeks. Conspicuous pores are benign, but this cosmetic concern represents a negative driver for the global skin beauty and quality perception, as well as a marker of skin aging.^2^, ^3^ Researchers aimed at describing the anatomical characteristics of pores and the factors influencing pore size and number.

Several endogenous and exogenous factors are said to be associated with pores: seborrhea, genetic predisposition, UV‐exposure, acne, or aging.^1^ Clinical observations confirmed that facial pores have multifactorial origin. A positive association between pore size and number with sebum output level, but not with acne, was confirmed by several studies.^4^, ^5^ Controversial data on the link between gender and pores have been reported and the latest results suggest that it is not the most critical factor.^4^, ^5^, ^6^ Enlarged pores increase with age, up to 30–40 years, and then stabilize or only slightly increase.^6^, ^7^ This rise could also be related to a modification of pores shape becoming more elongated with age.^2^, ^8^, ^9^ More than age, ethnicity impacts size and density of facial pores, with higher values observed in Indian and Brazilian populations.^6^ Another significant correlation was detected between R7 Cutometer® parameter, reflecting skin elasticity, and pore number in two independent studies suggesting that the structure of dermis could be involved in pore widening.^5^, ^10^ Other observations also support this idea: enlarged pore counts were related to wrinkle severity; and the loss of Microfibril‐associated glycoprotein‐1 in the hair follicle/pore area with aging and photo‐exposure, indicated a lack of structural support in the dermis.^7^, ^11^

Epidermal and dermal impairments were identified as an underestimated clinical cause of conspicuous pores.^6^ Microscopic imaging of pores area revealed inner structural changes affecting skin relief: a lower density of collagen in the deeper dermis, a thicker stroma and coarser collagen fibers forming a tubular structure around the follicle, an irregular dermo‐epidermal junction; that may result in an altered distribution of skin tensions.^12^, ^13^, ^14^, ^15^ The initial source of these alterations could be inflammatory phenomena, such as acne,^14^ and recent data suggest inflammaging, mediated by complement activation, as a possible player in the formation of enlarged facial pores.^16^ Defects in epidermal morphology around pores were also noted, as epidermis thickening and acanthosis, indicating aberrant keratinocyte proliferation.^13^ The presence of nucleated cells in the stratum corneum around pores suggests as well an abnormal differentiation of keratinocytes in the later stage of keratinization usually leading to corneocytes without organelles and nuclei.^17^

Treatment options to reduce conspicuous facial pores mainly target sebum reduction and collagen synthesis by using oral and topical retinoids, contraceptive pills and energy‐based devices (intense pulsed light, fractional ablative laser, non‐ablative laser, ultrasound or radiofrequency) but do not address all the morphological defects described above.^18^, ^19^ Topical anti‐aging were observed to significantly diminish visible pores but do not explore the mechanisms underlying this activity.^3^, ^18^, ^20^ Cosmetic products with characterized and effective pore refining activity could represent an alternative for users wishing non‐invasive solutions with no adverse effects.

In a previous work, mannose‐6‐phosphate was shown to stimulate fibroblasts proliferation and matrix remodeling; increase structural proteins and improve collagen fiber organization as well as reticular dermis density.^21^ A clinical trial confirmed mannose‐6‐phosphate increased collagen density in vivo and reduced wrinkles. In light of these results, mannose‐6‐phosphate appeared a potential pore‐refiner. The molecule was combined with amino acids, copper and lactic acid, a complex called Active Complex, that was evaluated in different models of stressed and altered dermis, as well as at the epidermal level to address causes of enlarged pores. Two clinical studies conducted on women and men, from different ethnical group, illustrated the pore refining activity of Active Complex.

## 2 MATERIALS AND METHODS

### 2.1 Active Complex composition

The composition was: sodium mannose‐6‐phosphate (2.5%–3.5%), D‐mannose (0.7%–1.7%), lysine (1.0%–1.4%), proline (0.7%–1.1%), copper sulfate (0.2%–0.4%), and lactic acid (0.8%–1.5%), 1,3‐propanediol (21%–29%) and its pH ranges from 4.5 to 5. Mannose‐6‐phosphate was obtained as previously described by the biocatalytic reaction between pyrophosphate and D‐mannose in the presence of acid phosphatase enzyme from *Shigella flexneri*.^21^ For easier reading, the composition is hereinafter mentioned as Active Complex.

### 2.2 Cells and skin source

All experiments were performed on primary cells and skin explants obtained from skin surgical residues following plastic surgery. Skin explants were obtained from donors who have sustained abdominoplasty and lifting (Polyclinique Courlancy, Reims) after reading, understanding and signing an “information and no objection” form for use, for dermocosmetic research purposes, of tissues, cells, and products of the human body collected during surgery (surgical residues), aligned with articles L. 1211‐2 alinéa 2, and L. 1245‐2, Code de la Santé Publique.

### 2.3 Study of active complex impact at the dermis level

#### 2.3.1 Gene expression study on a model of stressed fibroblasts mimicking premature aging

The cell culture was done on primary cells isolated from human skin biopsies. Normal Human Dermal Fibroblasts (NHDFs) were seeded at 300000 cells per well in 6‐wells plate. After 48 h of culture in Dulbecco's Modified Eagle Medium (DMEM) (Gibco, Life Technologies, CA, USA) with 10% fetal calf serum (FCS) (HyClone, USA) and 1% antibiotics (Sigma‐Aldrich, MI, USA) at 37°C and 5% CO_2_; cells were stressed with hydrogen peroxide (H_2_O_2_) at 200 μM for 2 h, to mimic premature aging versus basal condition. NHDFs were then rinsed two times with phosphate‐buffered saline (PBS) (Gibco, Life Technologies, CA, USA) and allowed to rest in medium FCS‐free overnight before stimulation. Cells were stimulated with Active Complex at 0.5% in premature aging condition. After 24 h of stimulation, total RNA was extracted by Extract‐all method (TriReagent, Invitrogen). RNA quality was controlled and a reverse transcription was performed to obtain cDNA (SimpliAmp™, Thermofisher). RT‐qPCR was made on CFX Touch (Bio‐Rad) with specific plates designed to study transcriptomic expression of different genes involved in dermis biology for NHDFs. The results of gene expression obtained with fibroblasts were normalized according to HydroxyMethylBilane Synthase (HMBS) and Glyceraldehyde‐3‐Phosphate Dehydrogenase (GAPDH). Only genes presenting a significant modification according to untreated control are presented.

#### 2.3.2 Collagen encoding genes expression on skin explants with weakened dermis

Human skin samples were collected immediately after surgery and stored at 4°C until processed. On the day following surgery and skin sample collection, hypodermis excision was performed and 8 mm diameter round biopsies were punched, stripped and included in nourishing matrix. The human skin explants were maintained in culture with NativeSkin® medium (Genoskin, France) at 37°C, 5% CO_2_, in an incubator during 6 days. The experiment was carried out in triplicate in the following conditions: untreated skin explants, skin explant treated by enzymatic degradation, skin explant treated enzymatic degradation and active complex 1%.

After 3 days of pretreatment, the enzymatic degradation of skin explants consisted in incubation with an enzymatic solution of collagenase (Sigma‐Aldrich, Merck, Germany) diluted in Hanks' Balanced Salt Solution (Dutscher, Bernolsheim, France) so as to obtain only partial digestion of the collagen. Then the explants were placed in the incubator for 3 days of posttreatment.

Total RNA was extracted from skin explants using TRIzol (Euromedex, France), followed by a purification step using chloroform (ThermoFisher, Massachusetts, USA) and isopropanol (Sigma‐Aldrich, Merck, Germany). The purified RNA was converted to cDNA using the High‐Capacity cDNA Reverse Transcription Kit (ThermoFisher, Massachusetts, USA). The qPCR analyses were set up using Sybr Green PCR Master Mix (ThermoFisher) and forward/reverse primers of genes encoding collagen I (COL1A1), collagen V (COL5A1), collagen VII (COL7A1) and collagen XVIII (COL17A1). The fluorescence was read by Applied Biosystems 7300 Real‐Time PCR System (ThermoFisher). The cycle threshold was converted in delta delta CT using the house‐keeping gene. The fold change was calculated with the formula 2 exponents‐delta delta CT (fold change). The control sample was fixed at 1 (skin explant treated by enzymatic degradation). The inhibition or activation of gene expression after treatment was evaluated.

#### 2.3.3 Collagen I and decorin expression on a model of stressed fibroblasts mimicking premature aging

NHDFs culture was done on primary cells isolated from biopsies. Cells were seeded in 96 wells black plate at 10000 cells per well in triplicate. The cells were incubated for 48 h in DMEM medium supplemented with 10% FCS and 1% antibiotics at 37°C with 5% CO_2_. At the end of the incubation, cells were stressed with H_2_O_2_ (Sigma‐Aldrich, Merck, Germany) at 200 μM for 2 h at 37°C with 5% CO_2_ and were then rinsed twice with PBS and incubated for 72 h in basal medium (DMEM medium without FCS) supplemented with 1% antibiotics containing Active Complex at 1% versus transforming growth factor beta (TGF‐β) (Sigma‐Aldrich, Merck, Germany) at 10 ng/mL + Vitamin C (Sigma‐Aldrich, Merck, Germany) at 20 μg/mL for the positive control of pro‐collagen I synthesis.

At the end of the culture, cells were fixed for 5 min with a paraformaldehyde (PAF) (Avantor, VWR, France) solution at 2% and were then permeabilized for 15 min with PAF solution at 2% supplemented with 0.5% Triton™ X100 (Sigma‐Aldrich, Merck, Germany). Nonspecific sites were saturated with 3% bovine serum albumin (BSA) (Sigma‐Aldrich, Merck, Germany) and primary antibodies targeting collagen I and decorin were incubated overnight at 4°C. Next day, cells were rinsed and incubated with secondary antibodies coupled to Alexa Fluor 488 (Invitrogen, MA, USA) for collagen I (Abcam, UK) and to Alexa fluor 568 (Invitrogen, MA, USA) for decorin (Abcam, UK). Fluorescent pictures and detection were automatically taken with PICO tool (Molecular Devices, CA, USA).

### 2.4 Study of Active Complex impact at the epidermis level

#### 2.4.1 Gene expression study on keratinocytes

Normal Human Epidermal Keratinocytes (NHEKs) culture was done on primary cells isolated from human skin biopsies. NHEKs were seeded at 300000 cells per well in 6‐wells plate. After 48 h of culture in Epilife complete medium (supplemented with HKGS growth factors and 1% antibiotics) (Gibco, Life Technologies, CA, USA), NHEKs were rinsed two times with PBS and allowed to rest in medium factors‐free overnight before stimulation. Cells were stimulated with Active Complex at 0.5%. After 24 h of stimulation, total RNA was extracted by Extract‐all method. RNA quality was controlled and a reverse transcription was performed to obtain cDNA. RT‐qPCR was made on specific plates designed to study transcriptomic expression of different genes involved in epidermis biology for NHEKs. Gene expression of target genes was normalized against two housekeeping genes: Eukaryotic Translation Initiation Factor 2B Subunit Alpha (EIF2B1) and ABL Proto‐Oncogene 1, Non‐Receptor Tyrosine Kinase (ABL1). Only genes presenting a significant modification according to untreated control are presented.

#### 2.4.2 Modeling keratinocyte proliferation disturbance in reconstructed human epidermis

Reconstructed human epidermis (RHE) were cultivated in an air‐liquid interface with an optimized EpiLife medium (Gibco) (confidential, property of QIMA) and were topically pre‐treated for 24 h with Active Complex at 1%. RHE were then stressed for 48 h with oleic acid at 2.5% in presence or not of treatment to induce epidermal alteration. RHE were then fixed in formaldehyde solution. Fixed samples were then dehydrated in successive ethanol baths of increasing concentrations before being embedded in paraffin.

Transversal sections were performed using a microtome (5 μm thickness, 2 sections per slide, 1 slide per RHE) and kept at room temperature until analyses. Tissue sections were deparaffinized and stained according to the standard protocol of HE staining. Briefly, sections were stained with hematoxylin, rinsed and stained with eosin. Sections were then rinsed and mounted in aqueous medium. To evaluate the disturbance of keratinocyte proliferation, the number of nuclei in the stratum corneum was counted on a defined length of RHE section.

Tissue sections were deparaffinized and incubated at 95°C in an unmasking solution to optimize antigen–antibody interaction. Slides were cooled down at room temperature in the same solution. After saturation with TBS‐Tween‐2% BSA, tissue sections were incubated overnight with the primary antibody solution directed against involucrin, the keratinocytes differentiation marker. After washing, binding sites recognized by the primary antibody were revealed by a secondary fluorescent antibody (GAR‐568). Labeled tissue sections were mounted with Prolong containing DAPI to color nuclei. Labeled tissue sections were observed using a ZEISS 710 confocal microscope. Images were captured and processed with ZEN software (objective lens ×20).

### 2.5 Clinical evaluation of Active Complex pore refining activity

#### 2.5.1 INCI formula of active cream and placebo

Active cream was formulated according to the composition described below. The ingredients composing Active Complex are highlighted in bold. Placebo cream corresponded at the INCI formula without Active Complex ingredients. Composition of active formula: AQUA/WATER, CETHYL ALCOHOL, GLYCERYL STEARATE, PEG‐75 STEARATE, CETEH‐20, STEARETH‐20, ISODECYL NEOPENTANOATE, **PROPANEDIOL, LACTIC ACID, LYSINE, MANNOSE, SODIUM MANNOSE PHOSPHATE, COPPER SULFATE, PROLINE**, PHENOXYETHANOL, 1.2‐HEXANEDIOL, CAPRYLYL GLYCOL, DIMETHICONE, FRAGRANCE.

#### 2.5.2 Impact of Active Complex on enlarged pores in Asian skin

A pre‐clinical study in double blind and placebo‐controlled conditions was carried out on 19 Asian men aged over 35 years old (mean age 42 ± 10 years old). A group of 10 men applied active cream and a group of 9 men applied placebo formula. Volunteers have been recruited according to inclusion criteria which are having visible enlarged pores on the face (a detailed list of inclusion and non‐inclusion criteria is presented in Table S2). All the subjects participating in the study gave their informed consent signed at the beginning of the study. The study followed and was in compliance with the tenets of the Declaration of Helsinki. Volunteers applied cream twice daily containing either 1% of Active Complex or placebo on the full face for 7 days.

The enlarged pore reduction was analyzed by ColorFace® (Newtone Technologies, Lyon) based on total pore area quantification.^22^ The ColorFace® is a 2D acquisition system able to perform multimodal and standardized pictures of the face. This device is equipped to a captor of 24 M pixel. There are various acquisition modes: Ultraviolet (UV) picture, without filter picture, cross polarized picture, 45° standard picture and 60° standard picture. Digital pictures of the face were done at days 0 and 7 to analyze total pore surface.

Facial sebum secretion at the skin surface was measured with Sebutape® method in the upper part of the nasolabial folds: a polymeric film is sealed to the area of investigation and lipids are trapped onto the polymer that becomes transparent to light. Seborrheic activity of the sebaceous gland was also compared to skin shine measured by ColorFace® system.

#### 2.5.3 Impact of Active Complex on enlarged pores in Caucasian skin

A clinical study in double blind and placebo‐controlled conditions was carried out on 33 women aged between 27 and 66 years old (mean age 44 years old). The 33 volunteers were divided in 2 groups with homogenous distribution depending on the age of volunteers to allow an evaluation of the whole cohort and also by range of age: 20–30 years old (17 volunteers) and over 50 years old (16 volunteers). Volunteers have been recruited according to inclusion criteria which were having visible pores and a lack of skin luminosity, together with presenting dull complexion on the face (a detailed list of inclusion and non‐inclusion criteria is presented in Table S1). All the subjects participating in the study gave their informed consent signed at the beginning of the study. The study followed and was in compliance with the tenets of the Declaration of Helsinki. Volunteers applied cream twice daily containing 1% of Active Complex on one half of the face and placebo on the other for 56 days. The enlarged pore reduction was analyzed by VisioFace® (Canfield Scientific, Inc, USA) based on total pore area quantification.^10^

VisioFace® consists of obtaining high resolution photographs of the three‐quarter face, in completely reproducible lighting conditions, in cross polarized light and diffuse light. The acquisitions were carried out with a high‐resolution camera. The lens used is a Nikkon 60 mm equipped with filter. Lighting was provided by two flash lights. The flash heads were fitted with filter slots to hold polarizing gel HN32 (Sarelec, Toulouse, France).

To obtain cross polarized light, the filter on the lens of the camera was positioned at 90° compared to the polarization of the filters of the flashes. The polarized light, emitted by the flashes and reflected by the skin of the face at the time of picture capture, was “cut” by the camera filter. These reflections did not appear on the photograph.

In the case of diffuse light photos, the filter on the lens of the camera was turned to 45° compared to the position for cross polarized light. The analysis was carried out by a specific software developed by Spincontrol (Tours, France). After the correction of the gradient and after the binarization of the pictures, the backgrounds were deleted using a threshold. The analysis was carried out on a determined area that is identical at each time of the study. Total pore area (mm^2^) extracted from pictures was calculated to measure the reduction of pore after applications of products and over time.

### 2.6 Statistical analysis

For in vitro and ex vivo studies, all results are presented as mean ± standard error of mean (SEM) of three independent triplicates. For all studies from in vitro to in vivo, a Shapiro Wilk test was used to verify whether the raw data followed the Gaussian Law. In case of Normally‐distributed data, the mean values were compared using either an unpaired or paired *t* test (≤2 groups) or One‐way ANOVA followed by post hoc test (≥2 groups). In case of non‐normally‐distributed data, a Wilcoxon (paired) or Kruskal–Wallis tests followed by a Mann–Whitney U (unpaired) test were used for paired data or unpaired data, respectively.

Whatever the used statistical test, we considered significant results as following ^#^ *p* < 0.1, *p < 0.05, **p < 0.01 and ***p < 0.001.

## 3 RESULTS

### 3.1 Impact of Active Complex on dermis restructuration

#### 3.1.1 Upregulation of genes involved in dermis structure of aged fibroblast

Pore enlargement is associated with a loss of collagen density and extracellular matrix proteins that are relevant signs of aging. Oxidative stress was triggered with H_2_O_2_ on fibroblasts to model collagen decrease and matrix metalloproteinase increase, observed during aging process.^23^ A transcriptomic analysis on targeted genes involved in dermis structure, matrix degradation and skin homeostasis revealed that the expression of four genes was significantly modified in stressed fibroblasts incubated with Active Complex: collagen type III alpha 1 chain (COL3A1) and matrix metallopeptidase 1 (MMP1), 8 (MMP8), and 13 (MMP13). The results are presented in Figure 1 and were expressed in comparison with H_2_O_2_ stressed condition and normalized with the average of most stable housekeeping genes.

**FIGURE 1:**
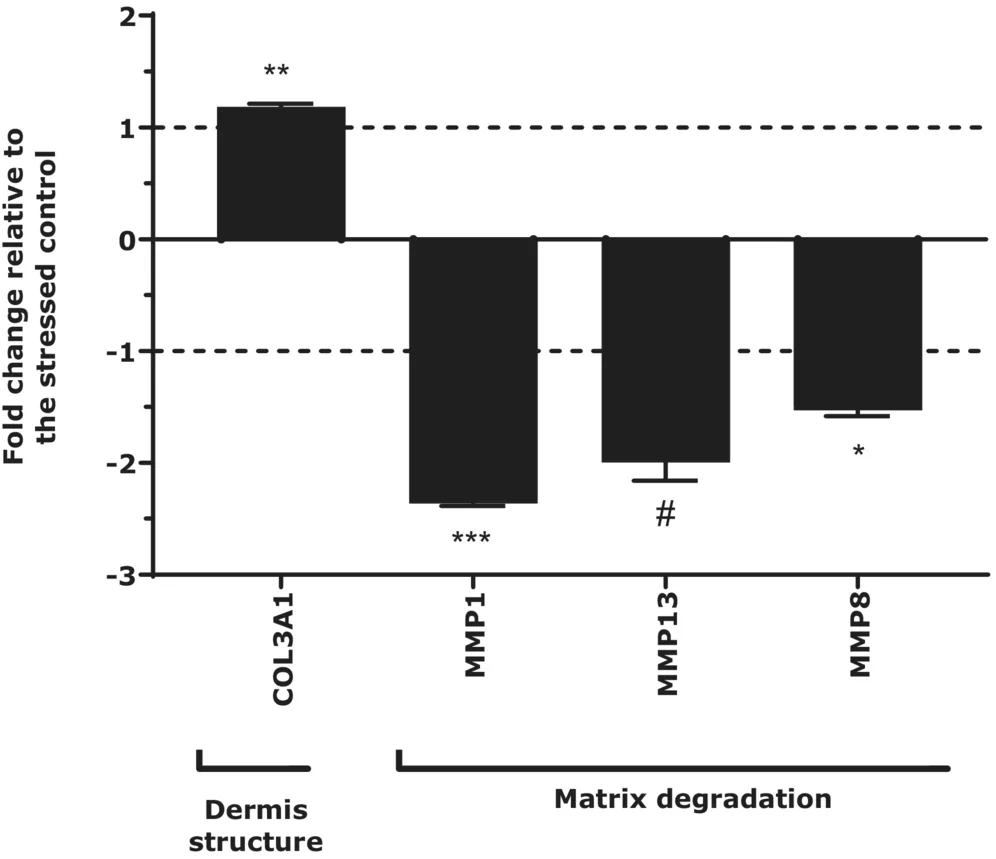
Regulation of five genes in fibroblasts treated with 0.5% Active Complex for, 24 h after induction of premature aging by H_2_O_2_. COL3A1 encodes collagen type III alpha 1 chain. MMP1, MMP13, and MMP8 encodes for matrix metallopeptidase 1, 8, and 13. The fold change was compared to H_2_O_2_ stressed control using Student's *t*‐test: #*p* < 0.1, *p < 0.05, **p < 0.01, and ***p < 0.001.

In the context of premature aging, Active Complex upregulated the expression of COL3A1 suggesting a stimulation of this protein that is abundant in the dermis and participates to extracellular matrix (ECM) structure and mechanical resistance of tissues.^24^ MMP1, MMP8, and MMP13 encode for collagenases capable of attacking native fibrillar collagen types I, II, and III.^25^, ^26^ The three genes were downregulated in stressed fibroblasts by Active Complex that could thus protect extracellular matrix proteins from degradation.

#### 3.1.2 Upregulation of collagen encoding genes in an ex vivo model of weakened dermis

The impact of Active Complex on collagen synthesis was also evaluated in ex vivo models presenting dermis alteration to mimic pore enlargement context. Skin explants were pre‐treated with Active Complex or distilled water (control) before induction of dermis weakening by enzymatic degradation of matrix components. The expression of genes encoding collagens I, V, VII, and XVII was analyzed by RT‐qPCR in control and treated conditions and expressed in percentage of the untreated condition (not exposed to enzymatic degradation treatment). Figure 2 presents gene expression of mRNA encoding collagens I, V, VII, and XVII in skin explants after enzymatic degradation of matrix components that significantly reduced collagens I, V, VII, and XVII expressions. Applying Active Complex on skin tissue restored the gene expression beyond the levels of the untreated control and confirmed the potential of Active Complex to promote collagen fiber synthesis, organization, anchorage, and cell cohesion.

**FIGURE 2:**
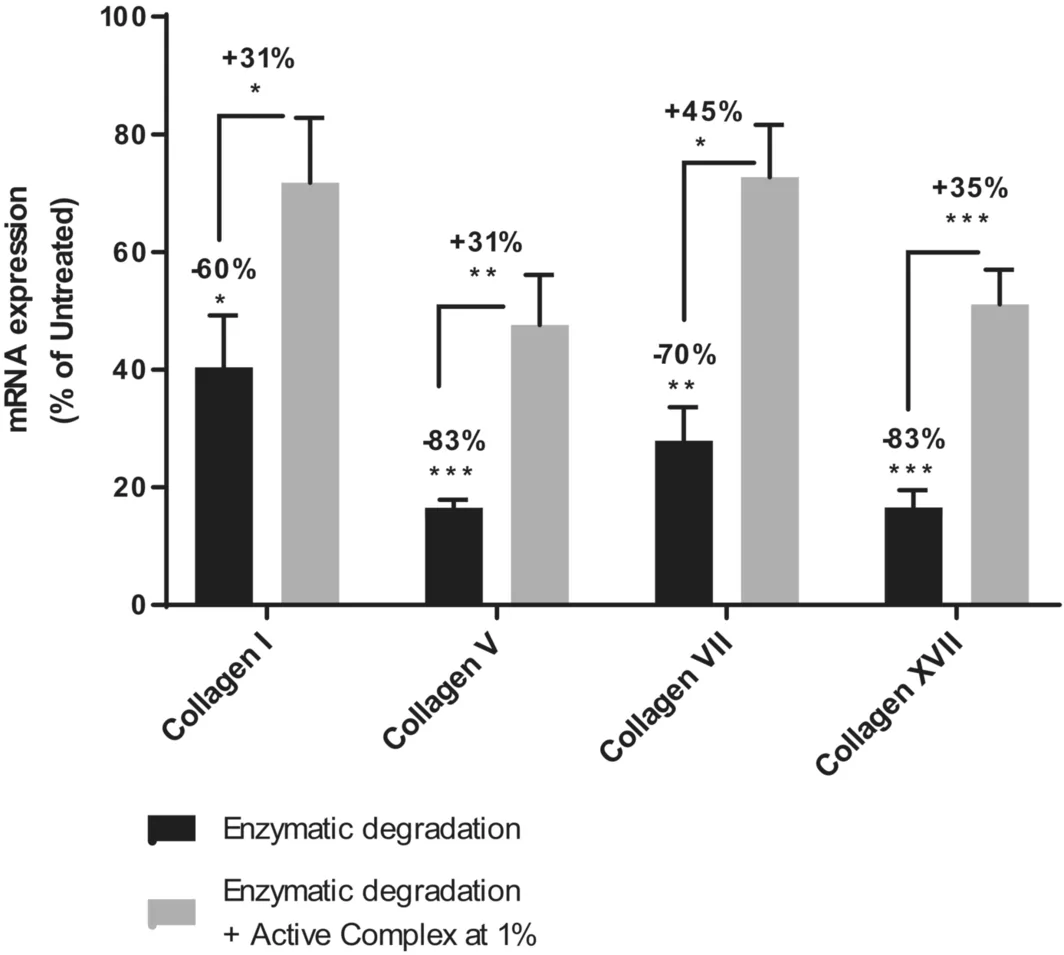
Gene expression of mRNA encoding collagens I, V, VII, and XVII in skin explants with weakened dermis induced by enzymatic degradation of matrix components. The fold change was expressed in percentage of the untreated condition (no dermis weakening) for the enzymatic degradation stressed condition (black bar); and expressed in percentage of the enzymatic degradation stressed condition for the condition treated with Active Complex (gray bar) using Mann–Whitney test: *p < 0.05, **p < 0.01, and ***p < 0.001.

#### 3.1.3 Stimulation of collagen I and decorin in aged fibroblasts

Besides the upregulation of collagen encoding genes, the ability of Active Complex to stimulate collagen fibrils was evaluated. collagen I and decorin, a proteoglycan playing a crucial role in collagen fibrillogenesis were quantified by immunofluorescence in fibroblasts after induction of a premature aging with hydrogen peroxide. TGF‐β and vitamin C were used as positive control of pro‐collagen I synthesis. Results are presented in Figure 3. Active Complex at 1% significantly stimulated the synthesis of collagen I fibrils (+22%) and also promoted decorin synthesis (+53%) at comparable levels of the positive control. Active Complex was able to improve collagen fibers organization within senescent fibroblasts.

**FIGURE 3:**
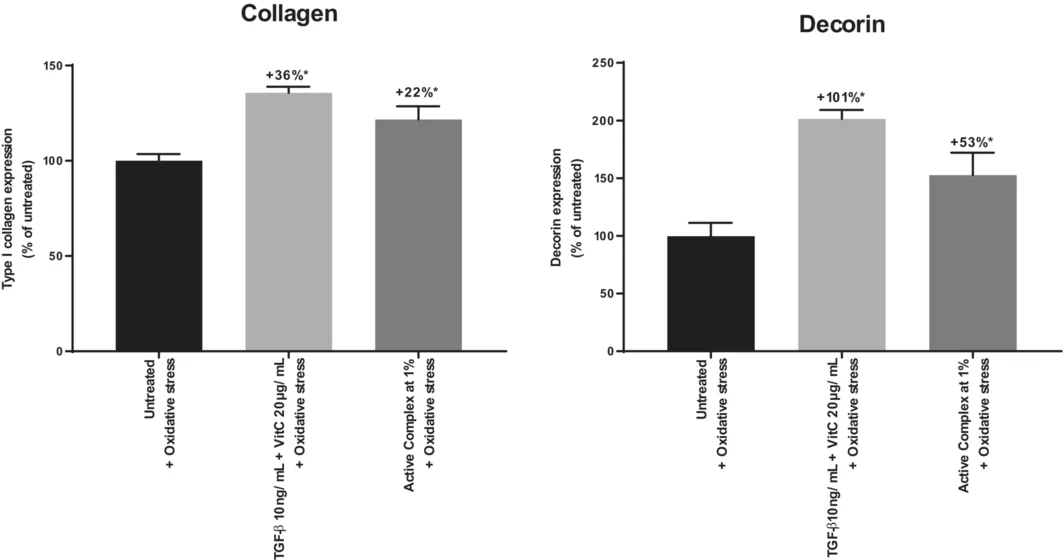
Quantification of collagen and decorin by immunofluorescence in fibroblasts‐induced premature aging with H_2_O_2_ (untreated), and treated with vitamin C and TGF‐β (positive control) or Active Complex 1%. Mann–Whitney test: *p < 0.05.

### 3.2 Impact of Active Complex at the epidermal level

#### 3.2.1 Active Complex regulates cell differentiation and renewal in keratinocytes

A transcriptomic analysis was performed to identify pathways regulated by Active Complex in NHEK. The analysis of transcripts was performed by RT‐qPCR targeting genes involved in desquamation, differentiation and stem cells markers. Results are presented in Figure 4. Three genes involved in epidermal organization were significantly downregulated: Loricrin (LOR) encoding a major protein of cornified cell envelopes, Small Proline‐Rich Protein 3 (SPRR3) related to cell proliferation and differentiation and corneodesmosin (CDSN) expressed in the upper layers of the epidermis. Taken together these data suggest that Active Complex could trigger a lowering of cornification process in keratinocytes.

**FIGURE 4:**
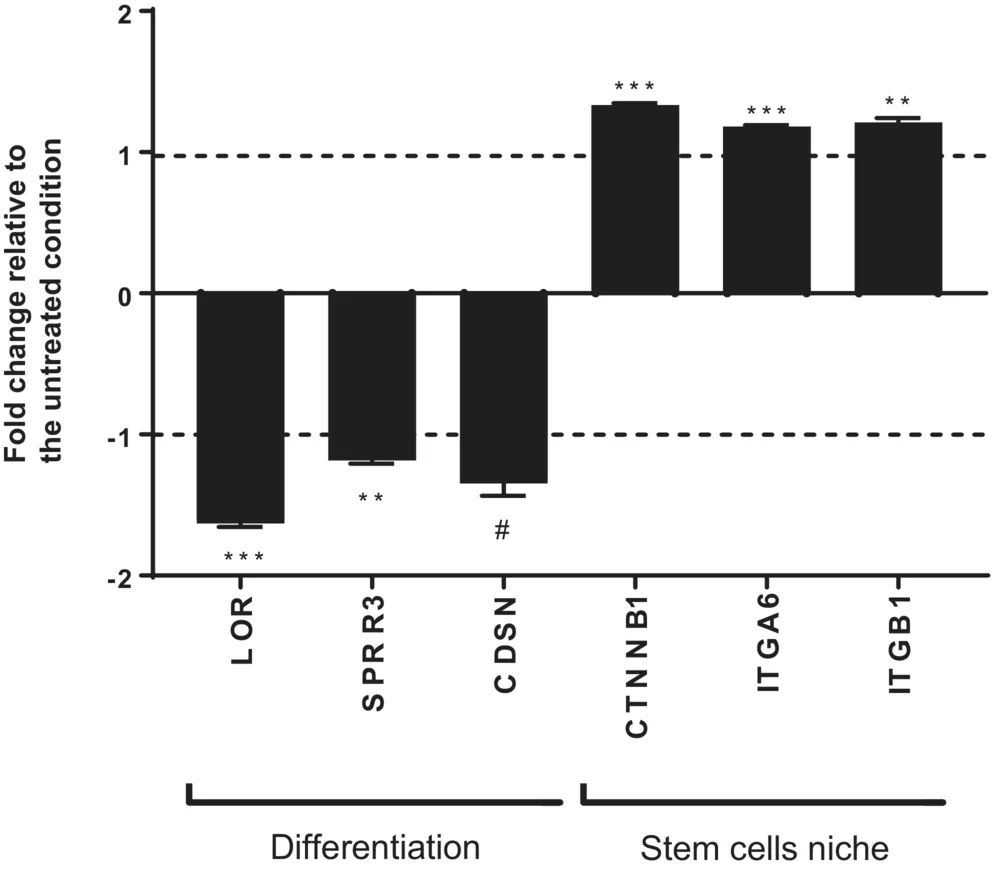
Regulation of six genes in keratinocytes (NHEKs) treated with Active Complex 0.5% for 24 h: Loricrin (LOR), Small Proline‐Rich Protein 3 (SPRR3), corneodesmosin (CDSN), Catenin (Cadherin‐Associated Protein) Beta 1 (CTNNB1), Integrin Alpha 6 (ITGA6) and Integrin Beta 1 (ITGB1). The fold change was compared to untreated NHEKs control using Student's *t*‐test: #*p* < 0.1, *p < 0.05, **p < 0.01, and ***p < 0.001.

The upregulation of Catenin (Cadherin‐Associated Protein) involved in the regulation of stem cell characteristics, beta 1 (CTNNB1) and Integrin Alpha 6 (ITGA6) involved in cell adhesion and communication and integrin beta 1 (ITGB1), a marker of undifferentiated keratinocytes, revealed the ability of Active Complex to positively impact cell renewal.

#### 3.2.2 In vitro modeling of keratinocyte hyperproliferation in RHE

Anatomical characterization of enlarged pores described a thickening of epidermis related to keratinocyte hyperproliferation.^12^, ^13^ It is also reported that nucleated corneocytes are more frequently observed around pores, suggesting an acceleration of keratinization process normally leading to anucleated cells.^17^ According to Katsuta et al., abnormal differentiation of epidermis was modeled by treating RHE with oleic acid at 2.5% for 48 h.^27^ Anatomical changes and involucrin expression were observed in untreated and stressed RHE and are presented in Figures 5 and 6 (untreated vs. stressed control). Involucrin was selected as a marker of keratinocyte differentiation.

**FIGURE 5:**
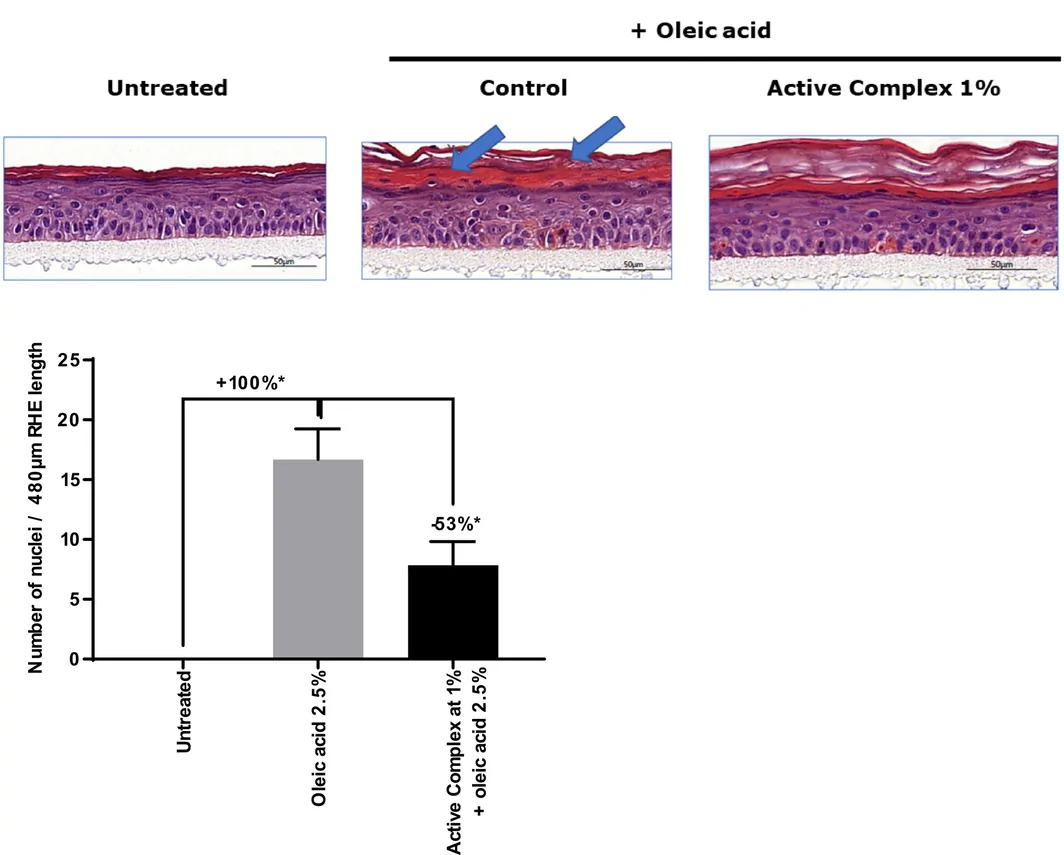
Up: Representative images of hematoxylin–eosin staining of RHE treated with 2.5% oleic acid with or without 1% Active Complex. Down: Quantification of nuclei in the stratum corneum. Comparison of normal RHE (untreated), RHE treated with oleic acid 2.5% (stressed control) and Active Complex 1%. Mann–Whitney test: *p < 0.05.

**FIGURE 6:**
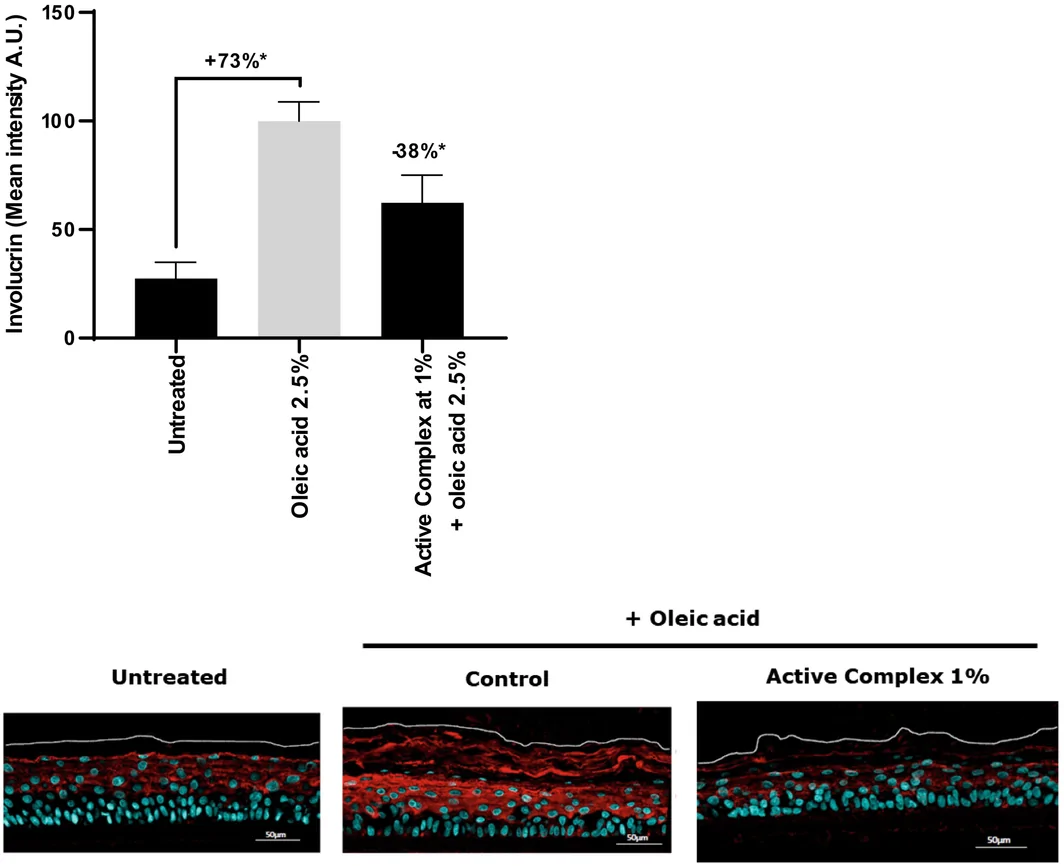
Up: Representative images of involucrin immunostaining treated with 2.5% oleic acid with or without 1% Active Complex. Down: quantification of the involucrin immunostaining mean intensity in RHE. Comparison of normal RHE (untreated), RHE treated with oleic acid 2.5% (stressed control) and Active Complex complex 1%. Mann–Whitney test: *p < 0.05.

Tissue treated with oleic acid presented a pathological morphology showing hyperkeratosis associated with spongiosis in the basal and spinous layers. In the untreated RHE, involucrin was expressed in the living layers of the epidermis according to a gradient from the basal to the granular layer while in oleic acid treated RHE the protein expression significantly increased especially in the stratum corneum. Besides the increase of involucrin expression, the number of nuclei significantly increased in the stratum corneum corroborating a dysregulation of epidermal differentiation process.

#### 3.2.3 Active Complex restored normal epidermal morphology

Active Complex was applied at 1% on RHE modeling keratinocyte hyperproliferation. Active Complex significantly reduced by 53% the number of nuclei in the stratum corneum compared to the stressed condition (oleic acid 2.5%) (Figure 5). Regarding involucrin, Active Complex significantly reduced by 38% the protein detection compared to the stressed control (Figure 6). Regarding morphology, the Active Complex significantly reduced spongiosis in the basal and spinous layers associated. Active Complex significantly reduced two markers of abnormal epidermal differentiation and restored a normal morphology.

### 3.3 Clinical evaluation of Active Complex as pore refiner

#### 3.3.1 Clinical study on Asian men cohort

This study was conducted on 19 Asian men volunteers over 35 years old (mean age 42 years), presenting conspicuous pores. Part of the group applied cream containing 1% of Active Complex in full face versus placebo for 7 days. The reduction of total pore area was measured with ColorFace® at days 0 and 7 (Figure 7). After 7 days of twice‐daily application, the total pore surface was significantly reduced by −12.6% and −30.2% in placebo and active‐treated group, respectively. The difference between the two groups were significant with a 2.4‐fold in favor of the group treated with Active Complex. These results confirmed the pore‐minimizing activity of Active Complex illustrated in Figure 8.

**FIGURE 7:**
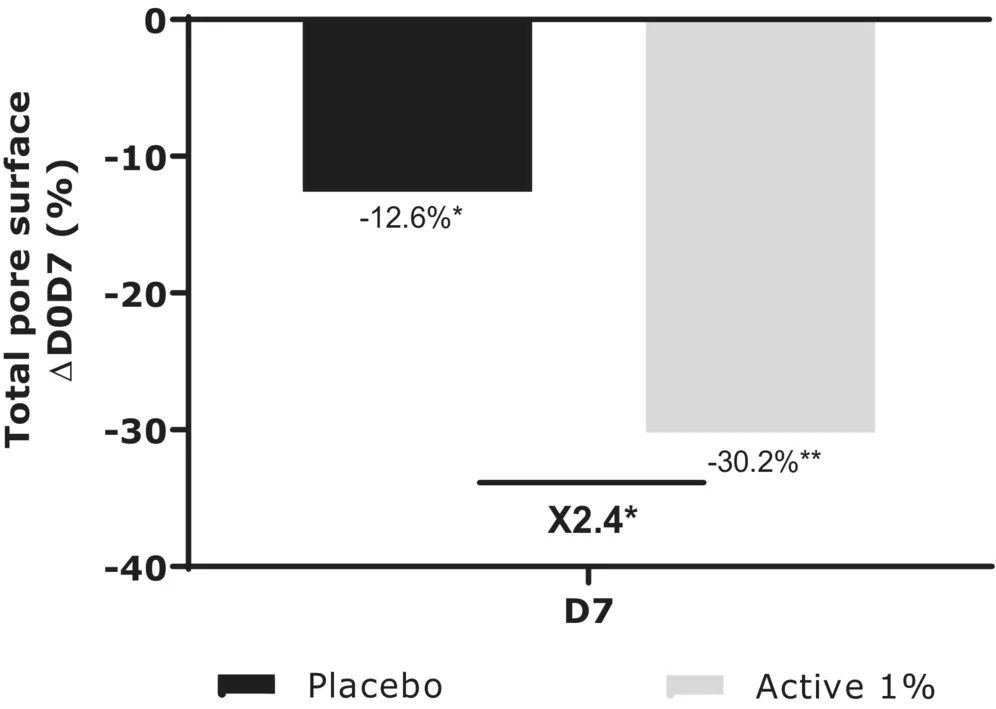
Comparison of total skin pore surface in placebo group and Active Complex treated group of the Asian men cohort after 7 days. Results are expressed in percentage of D0. Statistical significance is calculated using Student's *t*‐test: *p < 0.05 and **p < 0.01.

**FIGURE 8:**
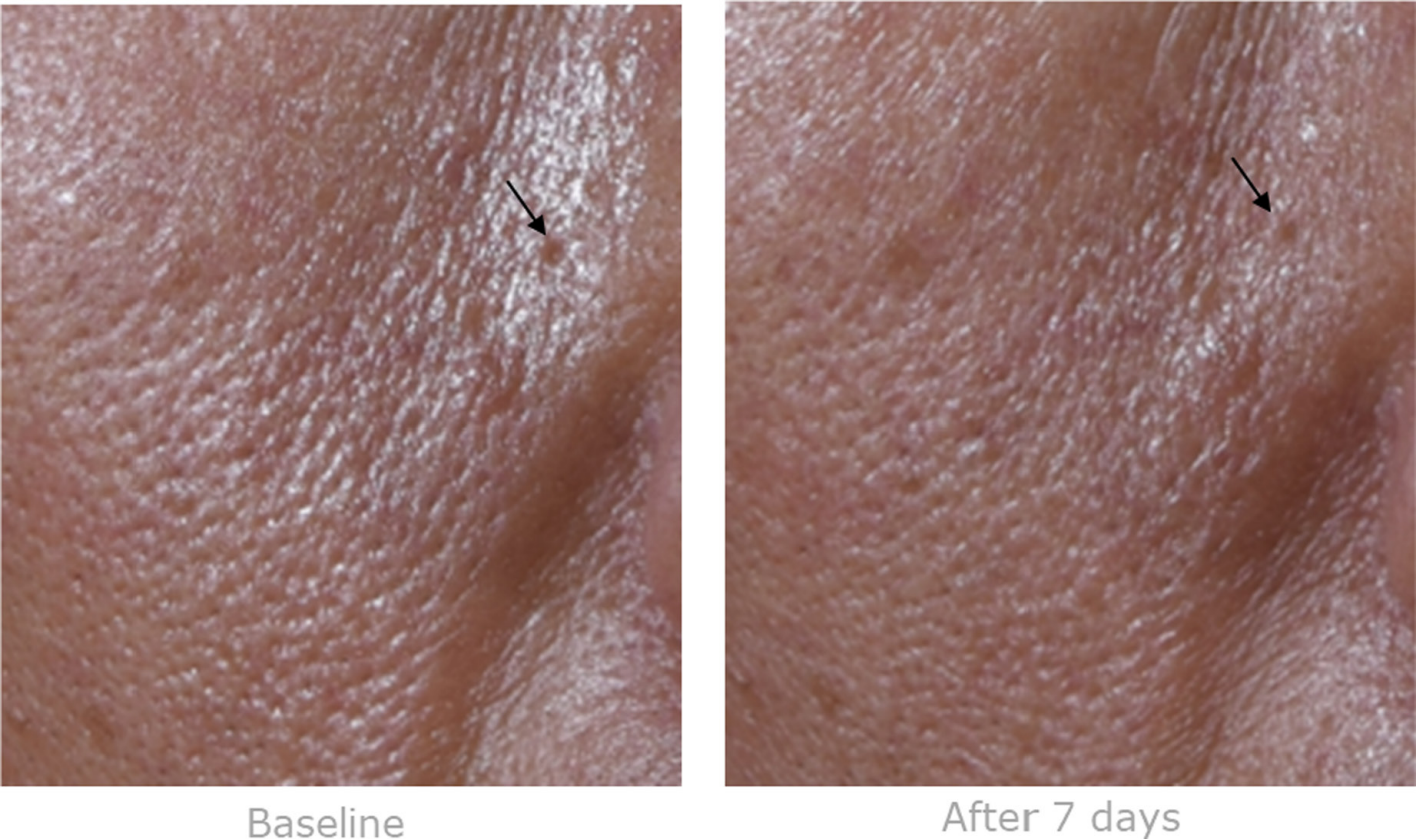
Illustrative picture of the pore refining activity of Active Complex after 7 days of application in an Asian men volunteer (60 years old). Arrow indicates a very large pore at day 0 with restricted surface at day 7.

An overproduction of sebum is often considered at the source of pore widening while some evidence refutes this statement.^6^ To clarify the mode of action of Active Complex, sebum production was evaluated on all volunteers by Sebutape® method (Figure 9). Skin shine, that depends on lipids secretion, was also measured by Colorface®. Sebum decreased similarly in both groups: placebo −5.5%, active −5.9%. This was confirmed by skin shine measurement that decreased by −18% and −21.5% in 7 days in placebo and active groups respectively and no significant difference was observed between the groups. This indicates that the slight sebum inhibition is assignable to the formula more than Active Complex and could not be related to its pore‐refining activity.

**FIGURE 9:**
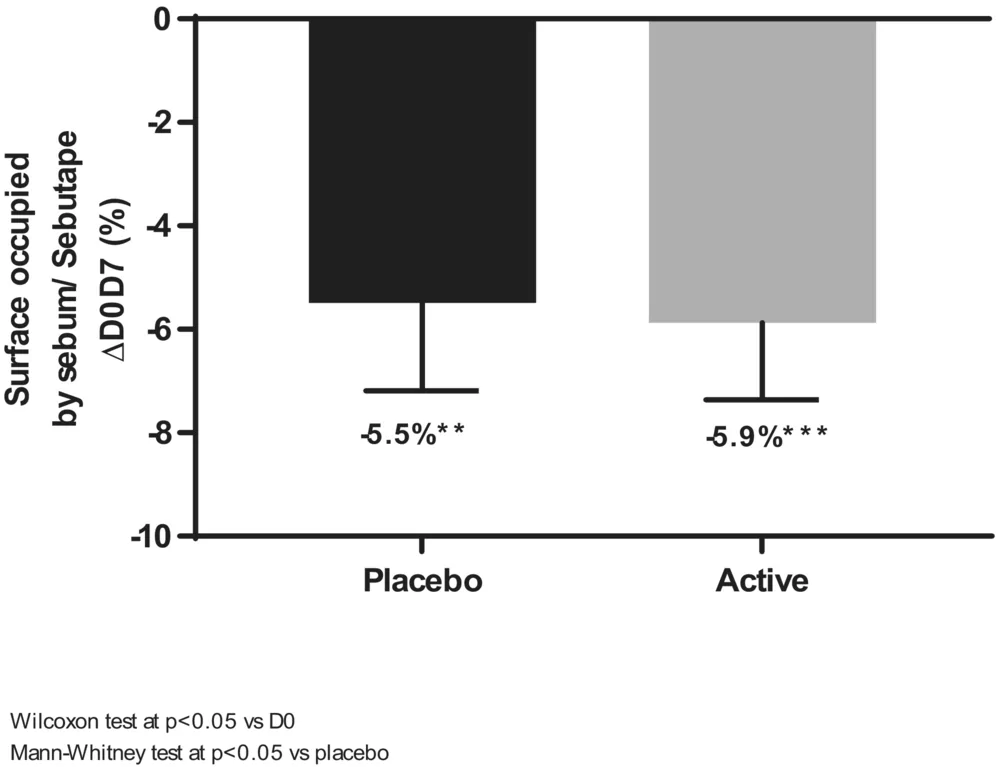
Comparison of seborrheic activity of sebaceaous glands in placebo group and Active Complex (Active) treated group of the Asian men cohort after 7 days. Results are expressed as a percentage of the surface occupied by skin lipids trapped on the sebutape® polymeric film versus day 0. Statistical significance is calculated from using Wilcoxon test versus D0 and Mann–Whitney test versus placebo: **p < 0.01 and ***p < 0.001.

#### 3.3.2 Clinical study on Caucasian women cohort

This study was conducted on women volunteers with enlarged pores applying cream containing 1% of Active Complex versus placebo in hemi face for 56 days. The reduction of total pore area was measured with VisioFace® at days 15, 28, and 56 (Figure 10) versus D0. The placebo formula had no impact on this parameter. From day 15, Active Complex reduced by −11.9% and reached −23.3% at day 56.

**FIGURE 10:**
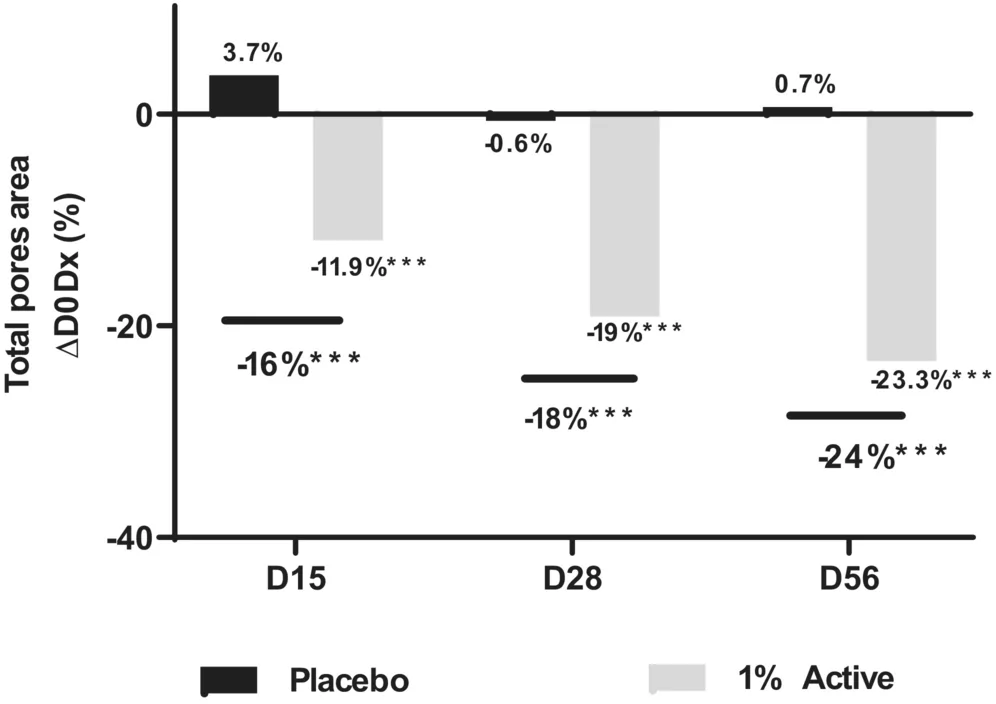
Total pore area quantification at days 15, 28, and 56 versus D0 or versus placebo in Caucasian women volunteers. Placebo formula and Active Complex 1% cream were applied in hemifaces. Statistical significance is calculated using Student's *t*‐test: ***p < 0.001.

Then the cohort was split into two groups according to age: young cohort (20–30 years old) (Table 1, Figure 11), and mature cohort (over 50 years old) (Table 2, Figure 12). At day 0, no significant difference was observed in the total pore are in young or mature women.

**TABLE 1:** Total pore area quantification at days 0, 15, 28 and 56 versus D0 or versus placebo in the young (20–30 years old) cohort of Caucasian women volunteers.

	Active Complex 1%			Placebo			
Day	Mean ± SD (arbitrary unit)	Average variation (%) vs. D0	Paired *t* test vs. D0	Mean ± SD (arbitrary unit)	Average variation (%) vs. D0	Paired *t* test vs. D0	Unpaired *t* test vs. placebo
**0**	9537 ± 3125			8283 ± 2935			
**15**	8901 ± 2983	−10.3%	*p* < 0.001 ***	8435 ± 3393	+3.7%	ns	*p < 0.05
**28**	7682 ± 3220	−19.5%	*p* < 0.001 ***	7491 ± 3051	−9.6%	**p < 0.01	*p < 0.05
**56**	7223 ± 2807	−24.3%	*p* < 0.001 ***	7809 ± 2361	−4.5%	ns	*p* < 0.001 ***

*Note*: *p < 0.05, **p < 0.01 and ***p < 0.001.

Abbreviation: ns, not significative.

**FIGURE 11:**
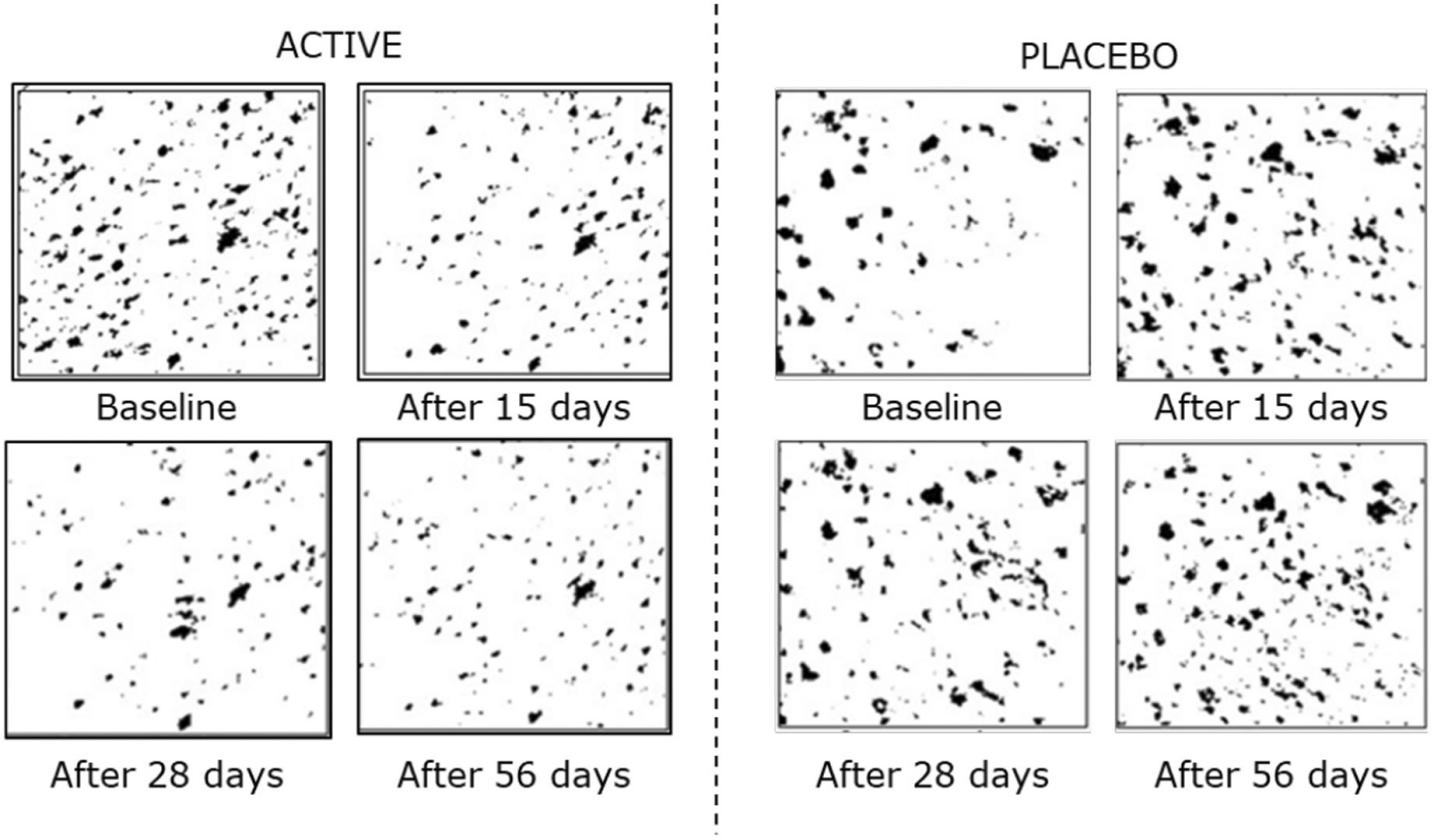
Illustrative images representing the automatic detection of skin pore surface of a volunteer from the young cohort of the Caucasian panel (29 years old) obtained with VisioFace®. On the right the squares in black and white are extracted from the hemiface treated with active formula (Active Complex) and on the left from the hemiface treated with placebo.

**TABLE 2:** Total pore area quantification at days 0, 15, 28 and 56 versus D0 or versus placebo in the mature (over 50 years old) cohort of Caucasian women volunteers.

Active Complex 1%				Placebo			
Day	Mean ± SD (arbitrary unit)	Average variation (%) vs. D0	Paired *t* test vs. D0	Mean ± SD (arbitrary unit)	Average variation (%) vs. D0	Paired *t* test vs. D0	Unpaired *t* test vs. placebo
**0**	8850 ± 3438			7987 ± 3984			
**15**	7645 ± 3532	−13.6%	*p* < 0.001 ***	8284 ± 3886	+3.7%	ns	**p < 0.01
**28**	7195 ± 3397	−18.7%	*p* < 0.001 ***	8680 ± 4074	+8.7%	ns	**p < 0.01
**56**	6877 ± 3542	−22.3%	*p* < 0.001 ***	8445 ± 4534	−5.7%	ns	*p* < 0.001 ***

*Note*: *p < 0.05, **p < 0.01 and ***p < 0.001.

Abbreviation: ns, not significative.

**FIGURE 12:**
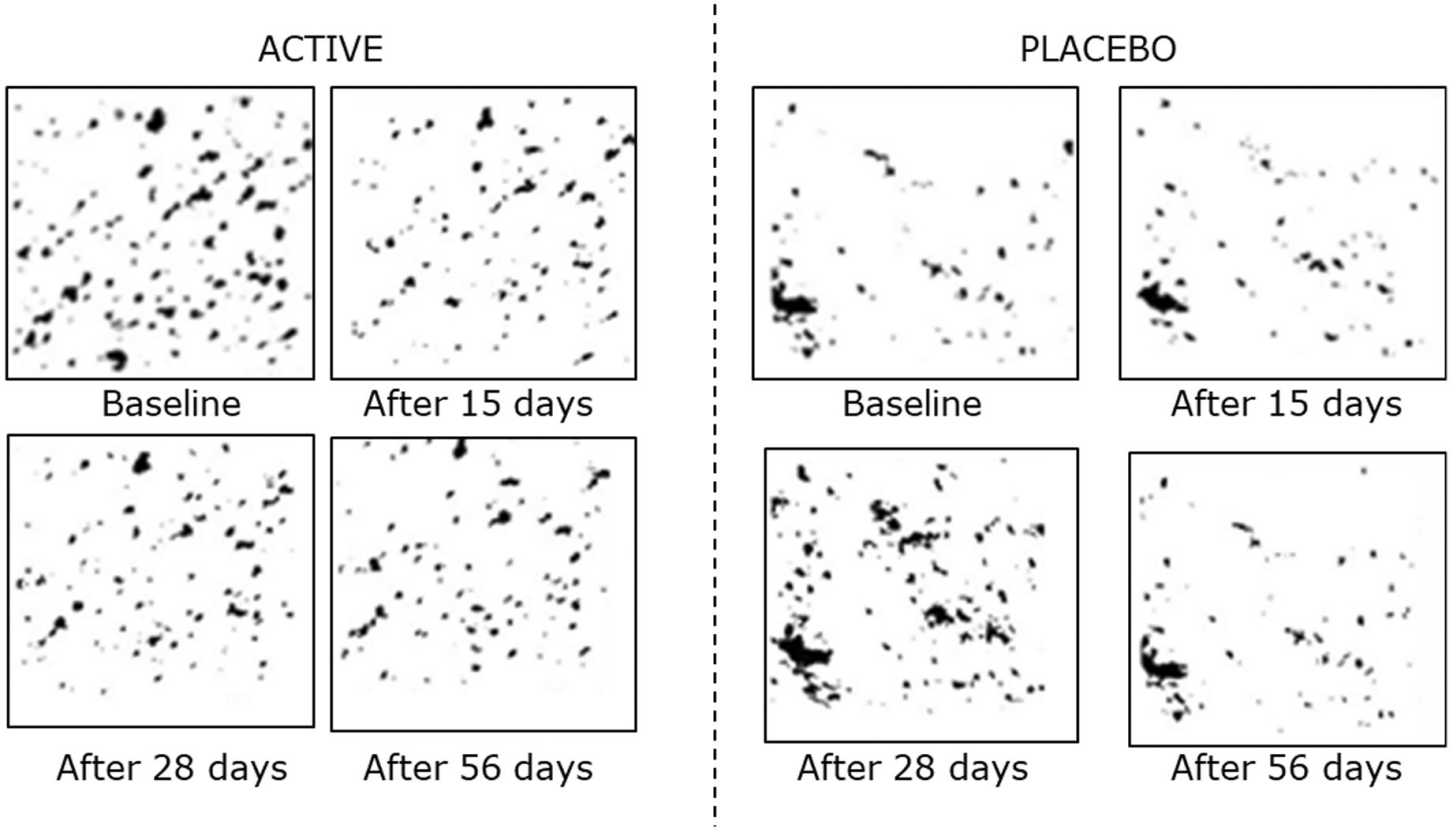
Illustrative images representing the automatic detection of skin pore surface of a volunteer from the mature cohort of the Caucasian panel (57 years old) obtained with VisioFace®. On the right the squares in black and white are extracted from the hemiface treated with active formula (Active Complex) and on the left from the hemiface treated with placebo.

In the young cohort, Active Complex reduced total pore surface from day 15 at −10.3% and reached −24.3% at day 56. The placebo did induce a significant reduction by −9.6% after 28 days but it was significantly different in comparison with the Active Complex‐treated area and in favor of the active. In the mature cohort we observed −13.6% reduction of pore surface area from day 15 that reached −22.3% after 56 days. Skin pore refining activity of Active Complex was similar in both groups.

## 4 DISCUSSION

Facial pores are the visible consequence of enlarged pilosebaceous follicles. Although benign, conspicuous pores are a beauty concern for men and women from puberty to mature age. Pore widening is often addressed by treating sebum overproduction whilst literature present dermal and epidermal impairments as two other relevant causes of this cosmetic defect and so are potential targets for the treatment of this cosmetic disorder.^6^, ^17^, ^28^, ^29^

In the pore area, dermis is subjected to modifications conducting to stroma alterations and causing pore enlargement: a tubular structure develops around pilosebaceous follicle, characterized by coarse collagen fibers replacing reticular collagen, and deeper dermis present lower density of collagen.^14^ Conspicuous pores are also associated to wrinkle severity and a possible loss of extracellular matrix protein, suggesting similarities with dermis weakening during aging.^7^, ^11^ At the epidermal level, enlarged pore's morphology present epidermis thickening and acanthosis as well as nucleated cells in the stratum corneum, suggesting aberrant keratinocyte proliferation.^13^, ^27^

Mannose‐6‐phosphate was previously demonstrated to promote dermis remodeling in the context of aging and improved biomechanical properties of the skin.^21^ The Active Complex, including mannose‐6‐phosphate, mannose, lysine, proline and copper was designed and evaluated in the treatment of visible pores. Amino acids were selected as main precursors of collagens that play a role in cell metabolism or collagen fibers formation. Lysine is a polar amino acid so it is present at the protein surface. In type I collagen, intricate post‐translational modifications of lysine residues initiate non‐enzymatic condensation reactions to form extensive covalent intra‐ and intermolecular crosslinks which are critical for the biomechanical functions of the collagen fibrils.^30^ Proline is particularly present in proteins of the extracellular matrix and collagen synthesis is highly dependent on this aminoacid.^31^ Copper was chosen to stimulate dermal fibroblasts proliferation and upregulate collagens (I, II, V) and elastin fibers components (elastin, fibrillins).^32^

Clinical evaluations showed the pore refining activity of Active Complex on men and women of different age and ethnicity. The first pre‐clinical study of Asian men cohort focused on the very short‐term effects of Active Complex on pore size. In 7 days, Active Complex decreased by −30.2% total pore surface. We observed significant effect which demonstrated the performance of the Active Complex considering a very short‐kinetic and inter‐individual variability. A second double blind and placebo‐controlled study was conducted on a larger cohort of Caucasian women with a longer time of application. Active Complex decreased total pore surface by −24.3% after 56 days of application, in both 20 and 30 years old and >50 years old volunteers. Although age and gender are not the most critical parameters affecting pores incidence, it is interesting to note that Active Complex was efficient independently of gender and age. Due to technical constraints, pore surface was assessed trough two different methods proved to rely on pore visibility: Visioface® and Colorface®.^10^, ^22^ This limits the comparison of results between ethnicities that deeply influence pore density and size.^6^ As an example, Caucasian and Japanese skin present similar density and surface of pores while Chinese skin are very less susceptible to this beauty defect. Nevertheless, Active Complex significantly decreased facial pores on both Asian and Caucasian skin, suggesting a robust mode of action. The assessment of facial sebum production revealed that Active Complex did not reduce seborrheic activity. It is unlikely that the efficacy of Active Complex in decreasing total pore surface would be linked to a regulation of sebaceous gland activity. Its mode of action at the dermal and epidermal levels were explored by in vitro and ex vivo experiments.

At the dermal level, Active Complex stimulated collagen I synthesis in senescent fibroblasts and in a fragile dermis context and increased COL3A1 gene expression. Collagen I and III are abundant in human skin representing 80% and 10% of total collagen, respectively.^33^ Collagen I is particularly localized in the reticular dermis, where a collagen decrease is observed in enlarged pores.^14^, ^24^ Active Complex increased decorin abundance, a protein contributing to fibrillogenesis. These data confirm the possible action of Active Complex on the renewal of collagen I fibers. The decrease of extracellular matrix protein degradation (MMP1, MMP8, MMP13) indicates that Active Complex also contribute to limit collagen depletion. A model of weakened dermis was established with skin explants enzymatically treated to induce dermis impairment, attested by a decrease of collagen I (60% vs untreated), collagen V (−83%), collagen VII (−70%), and collagen XVIII (−83%). Collagen V contributes to the lateral growth of collagens I and III fibers; while collagens VII and XVII plays critical role in skin stability and cohesion the one being a component of anchoring fibrils and the other being a transmembrane collagen, residing in hemidesmosomes.^33^ Active Complex restored gene expression of all these structural proteins (+31% to +45%). Active Complex seems able to induce the remodeling of collagen fibers within the dermis and responds to clinical cause of enlarged pores.

At the epidermal level, Active Complex firstly demonstrated an ability to downregulate genes involved in cell differentiation and to upregulate genes involved in stem cells niche. Using a model of RHE displaying keratinocytes hyper‐proliferation, Active Complex was shown to restore normal morphology and decrease the number of nucleated cells in upper layers of epidermis and involucrin expression, suggesting a slowdown of keratinization process that was observed to be deregulated in enlarged pores.

Pore enlargement origin is obviously multifactorial and the need of treatments with characterized mode of action would continuously improve the knowledge of this beauty concern. This work indicates Active Complex to treat two disregarded aspects of facial skin pores: on the one hand, dermis weakening by the slowdown of extracellular matrix proteases and the improvement of collagen fiber synthesis and organization; on the other hand the disturbance of epidermis differentiation by regulating keratinization process.

## 5 CONCLUSION

The present work discusses the issue of enlarged facial pores and potential treatments for this cosmetic concern. The causes of pore widening are numerous and include sebum overproduction, dermal impairments, and epidermal impairments and can be a concern for both men and women of across ages. In this context, Active Complex, including mannose‐6‐phosphate, amino acids (lysine and proline) and copper, was designed and evaluated for the treatment of visible pores. Active Complex reversed dermis weakening and epidermal differentiation disturbance, showing potential as a treatment for enlarged pores. Clinical evaluations showed that Active Complex decreased total pore surface in both short‐term and long‐term studies.

## AUTHOR CONTRIBUTIONS

*Conceptualisation*: R.R. and A.S. *Methodology*: M.M., E.C., and M.D.T. *Validation*: A.S. and D.A. *Investigation*: P.A. and A.D. *Writing—original draft preparation*: M.D.T. *Writing—review and editing*: M.D.T. and A.S. *Supervision*: D.A. *Project administration*: R.R. All authors have read and agreed to the published version of the manuscript.

## CONFLICT OF INTEREST STATEMENT

The authors declare not conflict of interest.

## ETHICS STATEMENTS

All procedures performed in studies involving human participants were in accordance with the ethical standards of the institutional and/or national research committee and with the 1964 Helsinki declara‐tion and its later amendments or comparable ethical standards.

## CONSENT

All participants provided written informed consent and a photo consent statement before starting the study. Written informed consent has been obtained from the patients to publish this paper.

## Supporting information


Table S1.
Table S2.

## Data Availability

The data that support the findings of this study are available from the corresponding author upon reasonable request.
