# Supplementary material for: Facial pore refining by targeting dermal and epidermal functions: Assessment across age and gender
Source: J Cosmet Dermatol. 2024 Jul 31;23(10):3395–408. doi: 10.1111/jocd.16424 (PMC13648923; doi:10.1111/jocd.16424)
Supplement: Supplementary file 1 [file JOCD-23--s001.docx]

**Supplementary material**

**Table S1: Inclusion and exclusion criteria for the study 1 “Impact of Active Complex on enlarged pores in Caucasian skin”**

| **Inclusion criteria** | **Non-inclusion criteria** |
| --- | --- |
| Female subject | Being or suspecting to be pregnant or wanting to get pregnant or breastfeeding |
| Healthy subject (no non-stabilized pathology likely to interfere with the study, no symptoms in the process of an exploratory checkup) | Having refused to give their agreement by not signing the informed consent form, |
| From 20 to 30 years (for 17 subjects), | Participating in another study liable to interfere with this study |
| More than 50 years (for 16 subjects), | Having started, changed or stopped a treatment for diabetes or for thyroid problems in the past 6 months |
| Caucasian subject | With a diagnosed allergy to one or several ingredients of cosmetic products or to |
| With healthy skin on the studied anatomic unit (free of eczema, psoriasis, wounds, inflammatory scar….) | Having applied a cosmetic product (including make-up) to the studied areas the first day of the study (the face must be cleaned only with water) |
| Having visible pores | Refusing to follow the restrictions below during the study:  - Do not become pregnant, do not want to get pregnant and do not breastfeeding,  - Do not participate in another study liable to interfere with this study  - *During the study*: It is prohibited to use other cosmetic product(s) than the test product(s) on the studied area(s) (only the usual cleanser, eyes and lips make-up as well as powder blush are allowed)  - *The day of the measurements*: It is prohibited to apply any cosmetic product to the test area (the face must be cleaned only with water) |
| Presenting a lack of skin luminosity, dull complexion on the face | Having undergone surgery on the studied area in the previous 6 months |
| Having fair skin (phototype I, II or III) | Having undergone techniques with aesthetic aim (chemical peel, mechanical dermabrasion, laser, pulsed flash lamp etc.) or having made injections of anti-wrinkle products (collagen, hyaluronic acid, botulinic toxin etc.) in the past 6 months. |
|  | Having applied products with an anti-wrinkle action (Retinoic acid, retinol, retinaldehyde, A.H.A. etc.) or for skin luminosity, in the past week. |
|  | Having a suntanned skin on the studied areas which could interfere with the evaluations of the study |
|  | Refusing to follow the restrictions below during the study:  - Do not use products or techniques or surgery with an anti-wrinkle action  - Do not expose to the sun (The subject is required to respect a strict photo-protection, in particular by using an adapted sunscreen in case of sun exposure) |

**Table S2: Inclusion and exclusion criteria for the study 2 “Impact of Active Complex on enlarged pores in Asian skin”**

| **Inclusion criteria** | **Non-inclusion criteria** |
| --- | --- |
| Asian male volunteer | Showing signs of recent and intense sun or UV exposure |
| Aged over 35 years | Having applied an anti-aging product less than a week before the 1st visit |
| With visible dilated pores | Washing of measurement areas other than with water on the morning of the study |
| Informed and consenting patients who have undergone a general clinical examination attesting to their ability to participate in the study | Having applied products: exfoliation, peeling, self-tanning, depigmenting or radiance treatment in an institute ... for less than a month on the face and mask for less than 2 weeks. |
| Having signed the consent form (Consent of participation) | Having performed cosmetic treatments at a dermatologist (laser, peeling, mesotherapy, injection, etc.) on the face during the last 6 months, |
| Healthy volunteers | Suffering from systemic diseases or any dermatosis likely to interfere with the study according to the investigator's assessment |
| Affiliated to a social security system |  |
| Who understand French language: francophone subjects able to read the documents presented to them and to join freely to what is explained to them. |  |
